# Supplementary material for: Feasibility, reproducibility and characteristics of coronary bifurcation type assessment by three-dimensional optical coherence tomography
Source: PLoS One. 2022 Feb 1;17(2):e0263246. doi: 10.1371/journal.pone.0263246 (PMC8806074; doi:10.1371/journal.pone.0263246)
Supplement: S1 Table — SD = standard deviation, *including staged PCI, LM, left main; LAD, left anterior descending; LCx, left circumflex; RCA, right coronary artery; MV, main vessel; SB, side branch; POT, proximal optimization technique; QCA, quantitative coronary angiography; Ref, reference; Min, minimum; OCT, optical coherence tomography. (DOCX) [file pone.0263246.s001.docx]

**S1 Table. Supplementary Table**

| Variable | Parallel type  (n = 30) | Perpendicular type  (n = 30) | P |
| --- | --- | --- | --- |
| Age, mean ± SD | 71.4 ± 7.6 | 71.0 ± 8.3 | 0.85 |
| Male sex, n (%) | 22 (73.3) | 24 (80.0) | 0.54 |
| Hypertension, n (%) | 25 (83.3) | 21 (70.0) | 0.36 |
| Dyslipidemia, n (%) | 23 (76.7) | 20 (66.7) | 0.39 |
| Diabetes mellitus, n (%) | 12 (40.0) | 15 (50.0) | 0.44 |
| Hemodialysis, n (%) | 0 (0) | 4 (13.3) | 0.11 |
| Former smoker, n (%) | 21 (70.0) | 16 (53.3) | 0.18 |
| Clinical presentation |  |  | 0.20 |
| Stable angina pectoris, n (%) | 8 (26.7) | 5 (16.7) |  |
| Unstable angina pectoris, n (%) | 1 (3.3) | 6 (20.0) |  |
| Old myocardial infarction, n (%) | 2 (6.7) | 1 (3.3) |  |
| Silent ischemia*, n (%) | 19 (63.3) | 18 (60.0) |  |
| Treated bifurcation, n (%) |  |  | 0.16 |
| LM | 13 (43.3) | 21 (70.0) |  |
| LAD | 11 (36.7) | 5 (16.7) |  |
| LCx | 3 (10.0) | 3 (10.0) |  |
| RCA | 3 (10.0) | 1 (3.3) |  |
| Medina classification, n (%) |  |  | 0.51 |
| (1,1,1) | 1 (3.3) | 0 (0.0) |  |
| (1,1,0) | 12 (40.0) | 10 (33.3) |  |
| (1,0,0) | 1 (3.3) | 0 (0.0) |  |
| (0,1,1) | 2 (6.7) | 1 (3.3) |  |
| (0,1,0) | 14 (46.7) | 19 (63.3) |  |
| Stent type, n (%) |  |  | 0.37 |
| BMX-J | 10 (33.3) | 14 (46.7) |  |
| Resolute integrity | 11 (36.7) | 5 (16.7) |  |
| Ultimaster | 4 (13.3) | 5 (16.7) |  |
| Synergy (Promus Premier) | 5 (16.7) | 6 (20.0) |  |
| Stent size, mm | 3.0 [2.5 – 3.5] | 3.5 [3.4 – 3.5] | 0.002 |
| Stent length, mm | 22.0 [18.0 – 26.0] | 21.0 [17.5 – 24.5] | 0.78 |
| Kissing balloon inflation |  |  |  |
| MV balloon size, mm | 2.9 [2.5 – 3.3] | 3.5 [3.0 – 3.5] | 0.0004 |
| SB balloon size, mm | 2.25 [2.0 – 2.5] | 2.6 [2.5 – 3.0] | 0.0002 |
| Rotational atherectomy, n (%) | 1 (3.3) | 5 (16.7) | 0.19 |
| Pre-dilatation, n (%) | 28 (93.3) | 29 (96.7) | 1.00 |
| Post-dilatation, n (%) | 29 (96.7) | 30 (100.0) | 1.00 |
| POT, n (%) | 11 (36.7) | 23 (76.7) | 0.004 |
| Distal recrossing, n (%) | 29 (96.7) | 27 (90.0) | 0.61 |
| Pre-procedural QCA |  |  |  |
| Proximal main vessel |  |  |  |
| Ref. lumen diameter, mm | 2.9 [2.4 – 3.2] | 3.2 [2.7 – 4.3] | 0.02 |
| Min. lumen diameter, mm | 2.2 [1.8 – 2.9] | 2.6 [2.2 – 3.6] | 0.04 |
| % Diameter stenosis | 14.0 [3.9 – 33.6] | 16.9 [7.1 – 35.7] | 0.71 |
| Distal main vessel |  |  |  |
| Ref. lumen diameter, mm | 1.8 [1.6 – 2.3] | 2.3 [1.8 – 2.7] | 0.01 |
| Min. lumen diameter, mm | 1.0 [0.8 – 1.4] | 1.4 [0.9 – 2.1] | 0.04 |
| % Diameter stenosis | 39.0 [21.1 – 58.2] | 28.3 [20.4 – 54.8] | 0.60 |
| Side branch |  |  |  |
| Ref. lumen diameter, mm | 2.1 [1.6 – 2.4] | 2.9 [2.3 – 3.4] | 0.0001 |
| Min. lumen diameter, mm | 1.7 [1.2 – 2.1] | 2.6 [1.9 – 3.1] | 0.0002 |
| % Diameter stenosis | 17.1 [9.4 – 27.2] | 10.2 [5.2 – 17.2] | 0.006 |
| Pre-procedural OCT analysis |  |  |  |
| Proximal ref. lumen area, mm^2^ | 7.4 [5.2 – 9.7] | 12.1 [9.0 – 13.0] | 0.0001 |
| Distal ref. lumen area, mm^2^ | 4.3 [3.4 – 5.8] | 6.1 [4.7 – 7.7] | 0.003 |
| Minimum lumen area, mm^2^ | 1.6 [1.0 – 2.0] | 2.5 [1.5 – 3.4] | 0.002 |

SD = standard deviation, *including staged PCI, LM, left main; LAD, left anterior descending; LCx, left circumflex; RCA, right coronary artery; MV, main vessel; SB, side branch; POT, proximal optimization technique; QCA, quantitative coronary angiography; Ref, reference; Min, minimum; OCT, optical coherence tomography.
