# Supplementary material for: Feasibility, reproducibility and characteristics of coronary bifurcation type assessment by three-dimensional optical coherence tomography
Source: PLoS One. 2022 Feb 1;17(2):e0263246. doi: 10.1371/journal.pone.0263246 (PMC8806074; doi:10.1371/journal.pone.0263246)
Supplement: Attachment — Submitted filename: Response to Reviewers.docx [file pone.0263246.s003.docx]

We thank the reviewers for carefully reading for our manuscript and for the reviewers’ comments, which have helped us to further improve the quality of our manuscript. Edited parts are highlighted with yellow marker in the revised manuscript.

-----------------------------------------------------------------------------------
Our responses to the reviewer #1’s comments are as follows:

-----------------------------------------------------------------------------------

**Reviewer #1: The authors reported the feasibility and reproducibility of online visual 3D-OCT assessment of bifurcation lesion. 3D-OCT bifurcation type is tentatively correlated with the distal bifurcation angle measured by 3D-QCA. This manuscript is well written and organized. However, following issues need to be addressed.
Comments:
1. It is unclear what will be the clinical implication of the bifurcation type. Debate persists regarding the optimal treatment of side branch bifurcation lesions including assessment of clinical significance. How can the information of bifurcation type before stenting help us to determine the treatment strategy in bifurcation lesion? Basically, it is not a valuable publication unless the authors demonstrate the clinical implication of this study. Please discuss about that.**

**Answer:**

We thank the reviewer for the valuable comments. We consider that the clinical implications of the 3D OCT bifurcation type are following; 1) prediction of the SB compromise after crossover stenting, 2) decision-making of the SB dilatation strategy, 3) decision-making of the 2-stent technique, 4) prediction of the strut coverage of the jailing strut at the SB ostium.

1) *prediction of the SB compromise after crossover stenting*.

As the description in the discussion, Watanabe et al., reported that the thinner carina-tip angle (<51 degree) was the independent predictor of the SB compromise^1)^. Unfortunately, the carina-tip angle cannot be measured on the OCT console system. Our 3D-OCT bifurcation type was distinguished by 51 degree and easy to assess on the OCT console. We consider that the carina-tip angle can be replace to the 3D-OCT bifurcation type. We investigated association between the 3D-OCT bifurcation type and SB compromise in the previous our registry (3D OCT bifurcation registry^2))^). As the result, the rate of SB compromise was 43.1% (31/72) in the parallel type and 9.6% (3/31) in the perpendicular type (p=0.001). We thought that the parallel type is likely to pinch SB due to the carina shift.

2) *decision-making of the SB dilatation strategy*

According to our investigation of the 3D OCT bifurcation registry, when kissing balloon inflation (KBI) is performed after crossover stenting, stent tends to be deformed largely in the perpendicular type rather than in the parallel type bifurcation^2)^. Moreover, it showed that the incidence of the incomplete stent apposition at SB ostium after KBI with confirmation of the distal recrossing in the parallel type was significantly smaller than that in the perpendicular type (7.4 ± 6.7 % vs 11.7 ± 8.8 %, respectively, p=0.0176). This data suggests that KBI might be suitable in the parallel type bifurcation.

3) *decision-making of the 2-stent technique*

The previous report showed that the 1-year MACE rate in the DK crush group was significantly lower than that on the Culotte group among patients with distal bifurcation angle ≥ 70°^3)^, which was classified as the perpendicular type bifurcation. Therefore, the information of bifurcation type (parallel or perpendicular) before stenting will help us to determine the 2-stent strategy in bifurcation lesions.

4) *prediction of the strut coverage of the jailing strut at the SB ostium*

Watanabe et al. found that bifurcation angle influences the neointimal coverage over DES struts in the side branch ostium at coronary bifurcation lesions^4)^. They reported that a significant negative correlation was found between the uncovered strut percentage at the bifurcation segment and distal bifurcation angles (r = − 0.41, P = 0.0024). Therefore, we think that the information of bifurcation type (parallel or perpendicular) before stenting helps us to predict uncovered strut percentage at the side branch ostium in bifurcation PCI.

We added the paragraph regarding “*Clinical significances of BA and 3D-OCT bifurcation type*” into the discussion as follows:

“*Clinical significances of the coronary BA have been discussed in previous numerical reports. Because of the association between the 3D-OCT bifurcation type and the distal BA, the information of 3D-OCT bifurcation type (parallel or perpendicular) before stenting might help us to determine the treatment strategy in bifurcation lesions.*”

“*Chen et al. reported that the 1-year MACE rate in the DK crush group was significantly lower than that in the Culotte group among patients with distal BA ≥ 70°^26)^, which was classified as the perpendicular type bifurcation. Moreover, it has been reported that a significant negative correlation was found between the uncovered strut percentage at the bifurcation segment and distal BA (r = -0.41, P = 0.0024)^24)^. With respect to the ISA including the jailing struts at the bifurcation segment after KBI, the distal rewiring into the jailed SB could reduce ISA in the parallel type bifurcation whereas ISA was not always reduced by distal rewiring in the perpendicular type^14)^.*”

**Comments:
2. The authors showed a correlation with the distal bifurcation angle measured by 3D-QCA. However, the clinical impact on bifurcation angle before stenting is limited. I believe that it is more important to acquire optimal projection detected by coronary CT or functional assessment using FFR/****FFRCT/QFR than the recognition of bifurcation angle or type before procedure.**

**Answer:**

Thanks for your comments. We agree that it is important to acquire optimal projection or functional assessment, however, we focused on the bifurcation appearance assessed by 3D OCT in the present study, which is simple and highly reproducible method.

In the present study, coronary CT examinations were not performed in all consecutive cases who underwent PCI. Therefore, we investigated a correlation between the bifurcation type and the bifurcation angles derived by 3D-QCA which is theoretically more precise rather than 2D-QCA^5,6)^.

**Comments:
3. The authors did not describe the difference in lesion characteristic between parallel type and perpendicular. Is there any differences in lesion characteristics such as distal recrossing or appropriate POT or KBI which is the predictor of side branch complication? Please clarify this.**

**Answer:**

Regarding reviewer’s comments, we showed the difference between the parallel and the perpendicular type in the supplementary table below. The incidence of left main bifurcation lesion tended to be higher in the perpendicular type than that in the parallel type (70.0% vs 43.3%). Therefore, the vessel size, stent size and balloon size used for kissing balloon inflation in the perpendicular type were significantly greater than those in the parallel type. These factors were potential confounders to predict the incidence of ISA at bifurcation. Therefore, we did not compare the incidence of ISA between the parallel type and the perpendicular type.

We added the supplementary table and the sentence in the limitation as follows:

“*We did not compare the ISA at bifurcation between the parallel type and the perpendicular type due to difficulties adjusting many confounders (Supplementary table).*”

**Supplementary Table**

| Variable | Parallel type  (n = 30) | Perpendicular type  (n = 30) | P |
| --- | --- | --- | --- |
| Age, mean ± SD | 71.4 ± 7.6 | 71.0 ± 8.3 | 0.85 |
| Male sex, n (%) | 22 (73.3) | 24 (80.0) | 0.54 |
| Hypertension, n (%) | 25 (83.3) | 21 (70.0) | 0.36 |
| Dyslipidemia, n (%) | 23 (76.7) | 20 (66.7) | 0.39 |
| Diabetes mellitus, n (%) | 12 (40.0) | 15 (50.0) | 0.44 |
| Hemodialysis, n (%) | 0 (0) | 4 (13.3) | 0.11 |
| Former smoker, n (%) | 21 (70.0) | 16 (53.3) | 0.18 |
| Clinical presentation |  |  | 0.20 |
| Stable angina pectoris, n (%) | 8 (26.7) | 5 (16.7) |  |
| Unstable angina pectoris, n (%) | 1 (3.3) | 6 (20.0) |  |
| Old myocardial infarction, n (%) | 2 (6.7) | 1 (3.3) |  |
| Silent ischemia*, n (%) | 19 (63.3) | 18 (60.0) |  |
| Treated bifurcation, n (%) |  |  | 0.16 |
| LM | 13 (43.3) | 21 (70.0) |  |
| LAD | 11 (36.7) | 5 (16.7) |  |
| LCx | 3 (10.0) | 3 (10.0) |  |
| RCA | 3 (10.0) | 1 (3.3) |  |
| Medina classification, n (%) |  |  | 0.51 |
| (1,1,1) | 1 (3.3) | 0 (0.0) |  |
| (1,1,0) | 12 (40.0) | 10 (33.3) |  |
| (1,0,0) | 1 (3.3) | 0 (0.0) |  |
| (0,1,1) | 2 (6.7) | 1 (3.3) |  |
| (0,1,0) | 14 (46.7) | 19 (63.3) |  |
| Stent type, n (%) |  |  | 0.37 |
| BMX-J | 10 (33.3) | 14 (46.7) |  |
| Resolute integrity | 11 (36.7) | 5 (16.7) |  |
| Ultimaster | 4 (13.3) | 5 (16.7) |  |
| Synergy (Promus Premier) | 5 (16.7) | 6 (20.0) |  |
| Stent size, mm | 3.0 [2.5 – 3.5] | 3.5 [3.4 – 3.5] | 0.002 |
| Stent length, mm | 22.0 [18.0 – 26.0] | 21.0 [17.5 – 24.5] | 0.78 |
| Kissing balloon inflation |  |  |  |
| MV balloon size, mm | 2.9 [2.5 – 3.3] | 3.5 [3.0 – 3.5] | 0.0004 |
| SB balloon size, mm | 2.25 [2.0 – 2.5] | 2.6 [2.5 – 3.0] | 0.0002 |
| Rotational atherectomy, n (%) | 1 (3.3) | 5 (16.7) | 0.19 |
| Pre-dilatation, n (%) | 28 (93.3) | 29 (96.7) | 1.00 |
| Post-dilatation, n (%) | 29 (96.7) | 30 (100.0) | 1.00 |
| POT, n (%) | 11 (36.7) | 23 (76.7) | 0.004 |
| Distal recrossing, n (%) | 29 (96.7) | 27 (90.0) | 0.61 |
| Pre-procedural QCA |  |  |  |
| Proximal main vessel |  |  |  |
| Ref. lumen diameter, mm | 2.9 [2.4 – 3.2] | 3.2 [2.7 – 4.3] | 0.02 |
| Min. lumen diameter, mm | 2.2 [1.8 – 2.9] | 2.6 [2.2 – 3.6] | 0.04 |
| % Diameter stenosis | 14.0 [3.9 – 33.6] | 16.9 [7.1 – 35.7] | 0.71 |
| Distal main vessel |  |  |  |
| Ref. lumen diameter, mm | 1.8 [1.6 – 2.3] | 2.3 [1.8 – 2.7] | 0.01 |
| Min. lumen diameter, mm | 1.0 [0.8 – 1.4] | 1.4 [0.9 – 2.1] | 0.04 |
| % Diameter stenosis | 39.0 [21.1 – 58.2] | 28.3 [20.4 – 54.8] | 0.60 |
| Side branch |  |  |  |
| Ref. lumen diameter, mm | 2.1 [1.6 – 2.4] | 2.9 [2.3 – 3.4] | 0.0001 |
| Min. lumen diameter, mm | 1.7 [1.2 – 2.1] | 2.6 [1.9 – 3.1] | 0.0002 |
| % Diameter stenosis | 17.1 [9.4 – 27.2] | 10.2 [5.2 – 17.2] | 0.006 |
| Pre-procedural OCT analysis |  |  |  |
| Proximal ref. lumen area, mm^2^ | 7.4 [5.2 – 9.7] | 12.1 [9.0 – 13.0] | 0.0001 |
| Distal ref. lumen area, mm^2^ | 4.3 [3.4 – 5.8] | 6.1 [4.7 – 7.7] | 0.003 |
| Minimum lumen area, mm^2^ | 1.6 [1.0 – 2.0] | 2.5 [1.5 – 3.4] | 0.002 |

SD = standard deviation, *including staged PCI, LM, left main; LAD, left anterior descending; LCx, left circumflex; RCA, right coronary artery; MV, main vessel; SB, side branch; POT, proximal optimization technique; QCA, quantitative coronary angiography; Ref, reference; Min, minimum; OCT, optical coherence tomography.

-----------------------------------------------------------------------------------
Our responses to the reviewer #2’s comments are as follows:

-----------------------------------------------------------------------------------

**Reviewer #2**:**The manuscript entitled “Feasibility, reproducibility and characteristics of coronary bifurcation type assessment by three-dimensional optical coherence tomography” investigates the possible role of 3D-OCT to assess bifurcation angles as compared to 3D-QCA.**

**Comments:
1. According to authors data, 3D OCT seems to be reliable for the detection of parallel and perpendicular bifurcation angles (BA). However,** **a binary classification of bifurcation angles into two types only might be poorly accurate and 3D OCT might be not accurate in identifying less overt angles** **(e.g 40-60°).**

**Answer:**

We thank the reviewer for the valuable comments. We think this point is very important and agree with the reviewer comments. According to the comment, we analyzed that the diagnostic accuracy of the 3D-OCT bifurcation type in three angle categories. The concordance rate for parallel type bifurcation in cases with a distal BA of < 40° (n = 12) was 91.7% (11/12) and that for perpendicular type bifurcation in cases with a distal BA of ≥ 60° (n = 22) was 77.3% (17/22), but that in cases with a distal BA of 40-59° (n = 26) was 61.5% (16/26). We added this point into results as follows:

*“Moreover, the concordance rate of 3D-OCT bifurcation types was 61.5% (16/26) in identifying BAs close to the cut-off value (40-59°), whereas it was 82.4% (28/34) in cases outside this range of distal BA.” was added in the results.*

However, a binary classification of bifurcation angles based on 3D-OCT bifurcation type can be easily performed in a short time and we can assess it as soon as the OCT/OFDI pullback was performed during PCI procedure. We think it is useful to know the angle quickly and easily.

**Comments:
2. Authors justify the use of 3D OCT for the BA as 3d OCT represents also a useful tool for stent optimization. However, in several centres also IVUS is used for this purpose. Please discuss the possible role of IVUS in such context.**

**Answer:**

We agree that IVUS is recognized as a useful tool for stent optimization. IVUS also might predict the side branch complication and the bifurcation angle. The previous report showed that the ‘‘eyebrow’’ sign was a powerful predictor of the side branch damage after stent implantation in the main vessel in bifurcation coronary lesions without plaque involving the side branch and that narrower angiographic angles were associated with the presence of the ‘‘eyebrow’’ sign (62º ± 23º vs. 76º ± 24º, p <0.05)^7)^. We cannot mention which of IVUS or OCT is superior to predict the bifurcation angle.

We added the sentences in the limitations as follows:

“*Use of IVUS and OCT is recommended for stent optimization during the bifurcation stenting in the current guideline^27)^. IVUS also might predict the side branch complication and the bifurcation angle. The previous report showed that the ‘‘eyebrow’’ sign associated with narrower angiographic angles was a powerful predictor of the SB damage after stent implantation in the MV^28)^. Further study to investigate the superiority between both modalities may be warranted.*”

**Comments:**

**3. As reported by the authors, 51° is the cut-off for side branch complications. It would be interesting to add any possible complications in the parallel and perpendicular BAs, as well as in those with angles > and <51°.**

**Answer:**

We thank the reviewer for the valuable comments. We consider that the clinical implication of the 3D OCT bifurcation type will be following except for the prediction of SB compromise; 1) prediction of the SB compromise after crossover stenting, 2) decision-making of the SB dilatation strategy, 3) decision-making of the 2-stent technique. Detail discussion was described previously in the answer for the reviewer #1’s comment. According to the reviewer’s comment, we added the following sentences in the discussion:

“*Clinical significances of the coronary BA have been discussed in previous numerical reports. Because of the association between the 3D-OCT bifurcation type and the distal BA, the information of 3D-OCT bifurcation type (parallel or perpendicular) before stenting might help us to determine the treatment strategy in bifurcation lesions.*”

“*Chen et al. reported that the 1-year MACE rate in the DK crush group was significantly lower than that in the Culotte group among patients with distal BA ≥ 70°^26)^, which was classified as the perpendicular type bifurcation. Moreover, it has been reported that a significant negative correlation was found between the uncovered strut percentage at the bifurcation segment and distal BA (r = -0.41, P = 0.0024)^24)^. With respect to the ISA including the jailing struts at the bifurcation segment after KBI, the distal rewiring into the jailed SB could reduce ISA in the parallel type bifurcation whereas ISA was not always reduced by distal rewiring in the perpendicular type^14)^.*”

**Comments:
4. Although the readership might be familiar with the topic, it would useful to add a figure explaining the three different BAs (distal, proximal and main vessel).**

**Answer:**

We thank the reviewer for his/her suggestion and we agree to add a figure explaining the three different BAs (distal, proximal and main vessel) as **Figure 3**.

**Figure 3.**

**References**

1. Watanabe M, Uemura S, Sugawara Y, Ueda T, Soeda T, Takeda Y, Kawata H, Kawakami R, Saito Y. Side branch complication after a single-stent crossover technique: prediction with frequency domain optical coherence tomography. *Coron Artery Dis* 2014;4:321-9.
2. Okamura T, Nagoshi R, Fujimura T, Murasato Y, Yamawaki M, Ono S, Serikawa T, Hikichi Y, Norita H, Nakao F, Sakamoto T, Shinke T, Shite J. Impact of guidewire recrossing point into stent jailed side branch for optimal kissing balloon dilatation: core lab 3D optical coherence tomography analysis. *EuroIntervention* 2018;13:e1785-e1793.
3. Chen SL, Xu B, Han YL, Sheiban I, Zhang JJ, Ye F, Kuwan TW, Paiboon C, Zhou YJ, Lv SZ, Dangas GD, Xu YW, Wen SY, Hong L, Zhang RY, Wang HC, Jiang TM, Wang Y, Chen F, Yuan ZY, Li WM, Leon MB. Comparison of double kissing crush versus Culotte stenting for unprotected distal left main bifurcation lesions: results from a multicenter, randomized, prospective DKCRUSH-III study. *Journal of the American College of Cardiology* 2013;61:1482-1488.
4. Watanabe M, Uemura S, Kita Y, Sugawara Y, Gryo Y, Ueda T, Soeda T, Okayama S, Okura H, Kume T, Saito Y. Impact of branching angle on neointimal coverage of drug-eluting stents implanted in bifurcation lesions. *Coronary artery disease* 2016;27:8:682-689.
5. Onuma Y, Girasis C, Aben JP, Sarno G, Piazza N, Lokkerbol C, Morel MA, Serruys PW. A novel dedicated 3-dimensional quantitative coronary analysis methodology for bifurcation lesions. *EuroIntervention* 2011;7:629-635.
6. Collet C, Grundeken MJ, Asano T, Onuma Y, Wijns W, Serruys PW. State of the art: coronary angiography. *EuroIntervention* 2017;13:634-643.
7. de Lezo JS, Medina A, Martín P, Novoa J, de Lezo JS, Pan M, Caballero E, Melián F, o Mazuelos F, Quevedo V. Predictors of ostial side branch damage during provisional stenting of coronary bifurcation lesions not involving the side branch origin: an ultrasonographic study. *EuroIntervention* 2012;7:1147-1154.
